# Adrenal masses in oncology patients

**DOI:** 10.1186/1470-7330-14-S1-O45

**Published:** 2014-10-09

**Authors:** Rodney H Reznek

**Affiliations:** 1 Imaging Department, St Bartholomew’s Hospital, London EC1A 7BE, UK

## 

In the general population, adrenal masses are demonstrated in 2-9% of CT scans. Eighty per cent of these are benign cortical adenomas. However, in patients with cancer, over 50% of detected adrenal masses prove to be metastases. Despite this the techniques for the evaluation of adrenal masses in patients with cancer should be similar to patients without cancer and incidentally discovered adrenal masses. Extensive published material is now available on the diagnostic performance of CT, MRI and PET in the characterization of adrenal masses. It must be borne in mind, however, that these techniques are usually evaluated for their ability to positively identify adenomas. Other adrenal pathology also occurs incidentally and it is essential that the radiologist should be familiar with the range of appearances of this pathology. Examples of this include phaeochromocytomas, cysts, myelolipomas, haemorrhage and granulomatous infections. Furthermore, although cross-sectional imaging techniques, particularly when modified specifically to evaluate indeterminate adrenal masses, can prove to have an excellent diagnostic performance (with remarkably high specificity), all have limitations. It is a consideration of the relative comparative strengths and weaknesses of each of these imaging modalities that informs the choice of technique and interpretation of the findings. This short presentation outlines the key points relating to the most widely used techniques for evaluating the adrenal mass in patients with cancer and the evidence for their role in patient management.
